# Organelle regulation of ferroptosis after intracerebral hemorrhage

**DOI:** 10.1016/j.redox.2026.104214

**Published:** 2026-05-21

**Authors:** Na Zhou, Yang Du, Yinuo Wang, Xingquan Zhao, Yi Ju

**Affiliations:** a Department of Neurology, Beijing Tiantan Hospital, Capital Medical University, Beijing, China; b China National Clinical Research Center for Neurological Diseases, Capital Medical University, Beijing, China; c Laboratory for Clinical Medicine, Capital Medical University, Beijing, China

**Keywords:** Intracerebral hemorrhage, Ferroptosis, Organelles, Mitochondria, Membrane contact sites

## Abstract

Intracerebral hemorrhage (ICH) is a devastating form of acute cerebrovascular disease characterized by high mortality and long-term disability, posing a substantial public health burden. Ferroptosis has emerged as a pivotal mechanism underlying secondary brain injury (SBI) after ICH, driven by reactive iron accumulation, amplification of lipid peroxidation (LPO) cascades, and disruption of cellular antioxidant defenses. Despite growing evidence supporting its involvement, the precise regulatory networks governing ferroptosis after ICH remain to be fully clarified. This review integrates recent advances to delineate how mitochondria, the endoplasmic reticulum (ER), lysosomes, lipid droplets (LDs), peroxisomes, the Golgi apparatus, and inter organelle membrane contact sites (MCSs) contribute to ferroptosis after ICH. We further summarize current therapeutic strategies targeting ferroptosis from an organelle-centered perspective and highlight its potential as a promising avenue for future intervention.

## Graphical abstract

**Figure:**
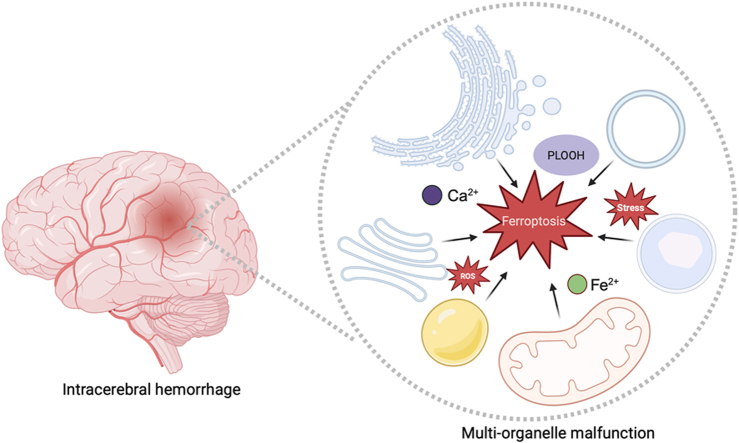

## 1 Introduction

Intracerebral hemorrhage (ICH) is defined as the rupture of a cerebral blood vessel followed by the entry of blood into the brain parenchyma. In 2021, stroke ranked the third leading cause of death with ICH accounting for 28.8% [1]. In terms of disability-adjusted life years (DALYs), ICH contributed nearly half of the stroke burden at 49.5%, while ischemic stroke (IS) accounted for 43.8% [2]. The pathophysiological mechanisms of ICH primarily include mechanical expansion of the hematoma, mass effect, and secondary brain injury (SBI). SBI begins within hours after ICH onset and progressively evolves over the following days to weeks, involving thrombosis, hemolysis, inflammatory response, oxidative stress, ferroptosis, blood brain barrier disruption, and cerebral edema [3]. Histopathologically, neurons may show cell shrinkage, nuclear condensation, cytoplasmic vacuolization, while glial and vascular cells exhibit reactive gliosis, inflammatory activation, phagocytic responses, and blood-brain barrier-related structural injury [4]. Human neuroimaging studies show that perihematomal iron deposition progressively increases and peaks around 30 days after ICH [5], while experimental models suggest that iron accumulation may persist for up to 12 weeks without complete clearance [6]. These findings imply that iron overload may contribute to prolonged brain injury after ICH. Ferroptosis is defined as an iron-dependent cell death characterized by lipid peroxidation (LPO) and eventual plasma membrane rupture [7,8], which is one of the primary forms of neuronal death after ICH [[9], [10], [11]].

The role of mitochondria in ferroptosis has been a major focus, whereas recent studies indicate that ferroptosis is not a single, isolated process but rather an integrated response involving multiple organelles. The endoplasmic reticulum (ER), lysosomes, lipid droplets (LDs), peroxisomes, and the Golgi apparatus also play key roles in iron homeostasis, LPO, and antioxidant defense systems. Recent work further indicates that membrane contact sites (MCSs) may serve as an initiating platform for ferroptosis and as inter-organelle conduits. In this review, we examine the mechanistic specific contributions to this process and further summarize advances in therapeutic strategies targeting ferroptosis in ICH from an organelle-centered perspective and highlight critical directions for future investigation.

## 2 Dynamic regulation of iron metabolism

**Fig. 1:**
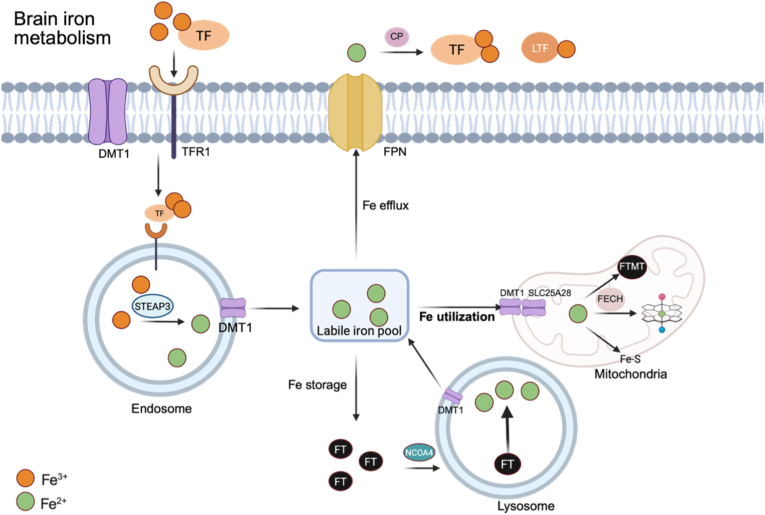
Main molecular mechanisms of Brain iron metabolism. TF-Fe^3+^ binds TfR1 and is internalized by endocytosis. In endosomes, Fe^3+^ is reduced by STEAP3 and exported to the cytosol as Fe^2+^ via DMT1, contributing to the LIP. Fe^2+^ in the LIP is trafficked by PCBP1/PCBP2 for storage, utilization or export. For storage, Fe^2+^ is oxidized and sequestered in ferritin nanocages; when iron demand rises, NCOA4-mediated ferritinophagy delivers ferritin to lysosomes to release Fe^2+^ back to the LIP. For export, PCBP2 channels Fe^2+^ to FPN; extracellularly, CP oxidizes Fe^2+^ to Fe^3+^ to reload transferrin. A fraction of the LIP is directed to mitochondria to support Fe–S cluster biogenesis, FTMT and heme synthesis. All figures are created in BioRender. zhou, N. (2026) https://BioRender.com/undefined. Abbreviations: TF, Transferrin; TfR1, transferrin receptor 1; DMT1, divalent metal transporter 1; STEAP3, six-transmembrane epithelial antigen of the prostate 3; LIP, labile iron pool; PCBP, poly(rC)-binding protein; FT, Ferritin; NCOA4, nuclear receptor coactivator 4; FPN, ferroportin; CP, ceruloplasmin; FECH, ferrochelatase; Fe-S, iron-sulfur; FTMT, mitochondrial ferritin; ISCU, Fe-S cluster scaffold protein; NFS1, the cysteine desulfurase; FXN, frataxin.

Under physiological conditions, systemic iron homeostasis is maintained primarily through dietary absorption in the intestine, followed by circulation in the bloodstream bound to transferrin and delivery to peripheral tissues [12,13]. Uptake of iron depends on transferrin receptor 1 (TfR1). Transferrin (TF) binds Fe^3+^ and engages TfR1 on the cell surface, after which the complex is internalized via endocytosis into endosomal compartments. Within endosomes, Fe^3+^ is released and reduced to Fe^2+^ by six-transmembrane epithelial antigen of prostate 3 (STEAP3), a metalloreductase located on the endosomal membrane. Divalent metal transporter 1 (DMT1) transports Fe^2+^ into the cytosol [14,15]. Additionally, heme catabolism also provides important supplementary sources of Fe^2+^. The cytosolic labile iron pool (LIP) constitutes the most bioavailable fraction of intracellular iron and is predominantly present in Fe^2+^ state. Ferritin is a hollow spherical nanocage assembled from 24 subunits and is primarily composed of heavy chain (FTH) and light chain (FTL) subunits [16,17]. Under iron demand, NCOA4 mediates ferritin delivery to autolysosomes for degradation, releasing Fe^2+^ back into the cytosol through ferritinophagy [18,19]. Conversely, under conditions of iron excess, cells limit iron accumulation by exporting Fe^2+^ through ferroportin (FPN), the only known transmembrane iron efflux transporter identified to date [13,20]. Iron efflux is further modulated by the peptide hormone hepcidin, which binds FPN and triggers its internalization and lysosomal degradation [21]. In the central nervous system, Fe^2+^ outside the plasma membrane is oxidized to Fe^3+^ by the ferroxidase ceruloplasmin (CP), enabling subsequent binding to TF for systemic transport [13,20,22,23]. Lactoferrin is an iron-binding glycoprotein secreted by neutrophils with a high affinity for Fe^3+^ [[24], [25], [26]]. A fraction of the LIP is directed into mitochondria to support the synthesis of essential iron containing cofactors. Fe^2+^ import into the mitochondrial matrix across the inner membrane is mediated by SLC25A37 (mitoferrin 1) and SLC25A28 (mitoferrin 2) [27]. Within the matrix, Fe^2+^ is predominantly utilized for the biosynthesis of iron-sulfur (Fe–S) clusters, heme and mitochondrial ferritin (FTMT). During heme synthesis, Fe^2+^ is inserted into protoporphyrin IX to generate heme, which is catalyzed by ferrochelatase (FECH) [28] (Fig. 1).

## 3 Hematoma resolution and ferroptosis after ICH

### 3.1 Hematoma resolution

After ICH, red blood cells (RBCs) break into the brain parenchyma and lead to oxidative modifications of membrane lipids and proteins. CD36 is a scavenger receptor responsible for recognition of damaged RBCs, which is upregulated by interleukin-10 [29,30]. RBCs failure to be phagocytosed are unstable and will spontaneously hemolyze and release hemoglobin (Hb) [31]. Haptoglobin (Hp) binds free Hb to form Hb-Hp complexes, which are subsequently recognized by CD163 expressed on the surface of phagocytes enters the lysosomal system, where it is degraded into heme and globin peptide fragments [32]. Owing to its lipophilic properties, free heme can readily intercalate into cellular membranes and injure neighboring cells. In brain tissue, hemopexin (Hpx) binds extracellular heme to form heme-Hpx complexes, which are subsequently internalized through low-density lipoprotein receptor-related protein 1 (LRP1), also known as CD91 [33]. Two heme oxygenase (HO) isoforms are found in the brain, where they catalyze the degradation of heme into biliverdin, carbon monoxide, and Fe^2+^. HO-1 is inducibly expressed after ICH in microglia, astrocytes, and other cell types, and constitutes an important enzymatic component of hematoma clearance. By contrast, HO-2 is constitutively expressed, predominantly in neurons [34,35]. The role of HO-1 after ICH, however, is complex and context dependent. Early HO-1 overexpression may exacerbate inflammation and brain edema, whereas its delayed upregulation appears to facilitate hematoma resolution and neurological recovery [36,37]. HO-1 also exhibits cell type-specific effects. In the early phase of ICH, microglial HO-1 overexpression may aggravate ferroptotic injury, whereas astrocytic HO-1 overexpression promotes blood-brain barrier repair and improves functional outcomes [38,39]. Moreover, whether other antioxidant components are concomitantly activated is also critical.

### 3.2 Ferroptosis after ICH

**Fig. 2:**
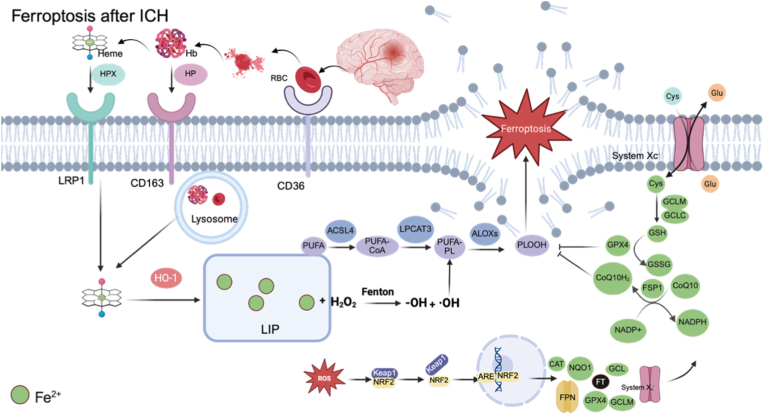
Ferroptosis after ICH. After ICH, RBCs and its degradation products enter phagocytes via CD36, CD163 and LRP1. HO-1 catabolize heme to expand the LIP. Excess Fe^2+^ drives Fenton chemistry to generate •OH, initiating lipid peroxidation. ACSL4 and LPCAT3 enrich membranes with PUFA-phospholipids that are oxidized by ALOXs, leading to PLOOH accumulation and ferroptosis. Antioxidant systems include NRF2/system Xc^−^/GSH/GPX4 axis and FSP1-CoQ10H_2_ axis. Abbreviations: CD36, cluster of differentiation 36; CD163, cluster of differentiation 163; LRP1, LDL receptor-related protein 1; HP, haptoglobin; HPX, hemopexin; HO-1, heme oxygenase 1; PUFA, polyunsaturated fatty acid; ACSL4, acyl-CoA synthetase long-chain family member 4; CoA, coenzyme A; LPCAT3, lysophosphatidylcholine acyltransferase 3; PL, phospholipid; ALOX, arachidonate lipoxygenase; •OH, hydroxyl radical; PLOOH, phospholipid hydroperoxide; NRF2, nuclear factor erythroid 2-related factor 2; KEAP1, kelch-like ECH-associated protein 1; ARE, antioxidant response element; CAT, catalase; NQO1, NAD(P)H quinone dehydrogenase 1; system Xc-, cystine-glutamate antiporter; Cys, cystine; Glu, glutamate; GCLC, glutamate-cysteine ligase catalytic subunit; GCLM, glutamate-cysteine ligase modifier subunit; GSH, reduced glutathione; GPX4, glutathione peroxidase 4; FSP1, ferroptosis suppressor protein 1; CoQ10, coenzyme Q10; CoQ10H2, reduced coenzyme Q10 (ubiquinol).

Beyond heme degradation, upregulation of TfR1 and DMT1 also promotes intracellular Fe^2+^ accumulation [40,41]. After ICH, elevated hepcidin suppresses FPN-mediated iron export [42]. Free Fe^2+^ can generate highly reactive hydroxyl radicals (•OH) via the Fenton reaction (Fe^2+^ + H_2_O_2_ → Fe^3+^ + OH^−^ + •OH) [7,8]. Long-chain acyl-CoA synthetase 4 (ACSL4) esterifies polyunsaturated fatty acids (PUFAs) to produce PUFA-CoA derivatives, which are subsequently incorporated into membrane phospholipids such as phosphatidylethanolamine (PE) and phosphatidylcholine (PC) through the action of acyltransferases including lysophosphatidylcholine acyltransferase 3 (LPCAT3) [43]. Members of the lipoxygenase family (ALOXs) function as important enzymatic amplifiers of LPO. ALOX5 promotes leukotriene biosynthesis and increases the overall LPO burden, while ALOX15 contributes to the oxidation of PUFA-PE [44]. •OH abstracts hydrogen atoms from PUFA-containing phospholipids (PUFA-PLs), generating phospholipid radicals (PL•) and initiating a self-propagating LPO cascade. PL• rapidly reacts with molecular oxygen to form phospholipid peroxyl radicals (PLOO•), which subsequently yield phospholipid hydroperoxides (PLOOH) and sustain chain propagation [45]. Progressive accumulation of PLOOH and related oxidized lipid species disrupts membrane architecture, ultimately resulting in plasma membrane rupture and ferroptosis cell death [45] (Fig. 2). The ultrastructural features of ferroptosis observed by transmission electron microscopy (TEM) include mitochondrial shrinkage, increased mitochondrial membrane density, reduced or vanished cristae, and rupture of the outer mitochondrial membrane [[9], [10], [11]].

### 3.3 Anti-ferroptosis system after ICH

Nuclear factor erythroid 2-related factor 2 (NRF2) is a master transcriptional regulator of cellular redox homeostasis and iron metabolism [46]. Kelch-like ECH-associated protein 1 (KEAP1) is a negative regulator of NRF2, promoting the ubiquitination and subsequent degradation of NRF2 [46]. When exposed to oxidative stress or electrophilic agents, KEAP1's ubiquitinating capacity diminishes, inhibiting NRF2 degradation and leading to its accumulation. Subsequently, NRF2 translocates into the nucleus to bind to the antioxidant response elements (AREs), and initiates the expression of antioxidant genes (e.g., NQO1, HO-1, GCLC, GCLM, FTH, FTL, CAT, etc.) [46] (Fig. 2). Peroxisome proliferator-activated receptor-gamma (PPARγ), a ligand activated member of the nuclear receptor superfamily, cooperates with NRF2 to suppress ferroptosis after ICH [47]. System Xc^−^ is a cystine and glutamate antiporter located on the plasma membrane. It consists of two subunits. Solute carrier family 7 member 11 (SLC7A11; xCT) mediates transport activity. Solute carrier family 3 member 2 (SLC3A2) supports protein stability and membrane localization. Via System Xc^−^, extracellular cystine is imported in exchange for intracellular glutamate, then reduced to cysteine, a precursor for glutathione (GSH) synthesis [48]. Glutathione peroxidase 4 (GPX4) serves as the principal enzyme that detoxifies membrane PLOOH. By utilizing GSH as a reducing equivalent, GPX4 reduces PLOOH to the corresponding phospholipid alcohol (PLOH) to interrupt the propagation of LPO [49,50]. After ICH, glutamate levels are elevated in perihematomal brain tissue [10,51,52]. Excess extracellular glutamate may inhibit system Xc^−^-mediated cystine uptake [53], thereby impairing the GSH-GPX4 antioxidant defense system. With hemin simulation, an increase in GPX4 and Nrf2 mRNA, accompanied by elevated GSH levels, was detectable after 6 h [54]. However, in a rat ICH model, GPX4 expression significantly decreased 12 h post-ICH and reached its lowest point at 24 h [55]. Taken together, these findings suggest that an early, compensatory antioxidant response occurs, but under continuous pathological stimulation, it ultimately breaks down. Ferroptosis suppressor protein 1 (FSP1, also known as AIFM2) is a newly identified key protein against ferroptosis, constituting a defense pathway independent of GPX4 [56,57]. FSP1 reduces oxidized CoQ10 to CoQ10H_2_ with NAD(P)H-dependent oxidoreductase. The highly lipophilic CoQ10H_2_ freely diffuses within biological membranes, directly scavenging LOO• and terminating the chain reaction of LPO [57,58]. After ICH, FSP1 expression was reduced, whereas omarigliptin (MK3102) restored FSP1 expression and concomitantly increased GPX4 [59].

## 4 The role of organelles in ferroptosis after ICH

### 4.1 Mitochondria

#### 4.1.1 Fe^2+^ and Ca^2+^overload and mitochondrial energy crisis

**Fig. 3:**
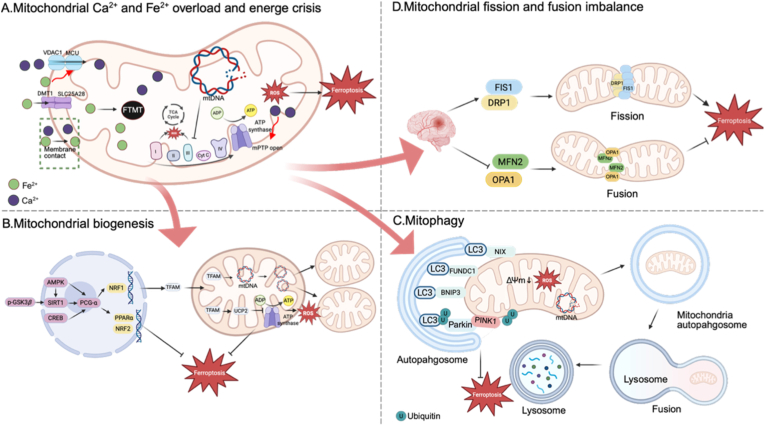
Mitochondrial responses linked to ferroptosis after ICH. (A) Mitochondrial Ca^2+^ and Fe^2+^ overload and energy crisis. After ICH, excess iron enter mitochondrial via SLC25A28 and membrane contact sites. MCU-mediated Ca^2+^ influx promotes matrix Ca^2+^ overload and mPTP opening. Fe^2+^ and Ca^2+^overload leads to increased ROS, mtDNA damage, impaired oxidative phosphorylation and ATP depletion. (B) Mitochondrial biogenesis. Mitochondrial biogenesis is coordinated by the SIRT1-PGC-1α axis, with AMPK, CREB and *p*-GSK3β included upstream. Activation of PGC-1α supports mitochondrial renewal and engages NRF2 and PPARα to defend against ferroptosis. UCP2 limits mtROS generation by uncoupling oxidative phosphorylation from ATP synthesis. (C) Mitophagy. Mitochondrial depolarization (ΔΨm loss), ROS and mtDNA injury cause mitophagy. Mitophagy is carried out by ubiquitin-dependent pathway and receptor-mediated pathways. (D) Mitochondrial fission and fusion imbalance. FIS1 promotes DRP1 recruitment to drive fission. Fusion is mediated by MFN2 and OPA1. Abbreviations:SLC25A28, solute carrier family 25 member 28 (mitoferrin 2); VDAC1, voltage-dependent anion channel 1; MCU, mitochondrial calcium uniporter; mPTP, mitochondrial permeability transition pore; mtDNA, mitochondrial DNA; SIRT1, sirtuin 1; PGC-1α, peroxisome proliferator-activated receptor-γ coactivator 1α; AMPK, AMP-activated protein kinase; CREB, cAMP response element-binding protein; *p*-GSK3β, phosphorylated glycogen synthase kinase 3β; NRF1, nuclear respiratory factor 1; TFAM, mitochondrial transcription factor A; NRF2, nuclear factor erythroid 2-related factor 2; PPARα, peroxisome proliferator-activated receptor-α; UCP2, uncoupling protein 2; mtROS, mitochondrial reactive oxygen species; ΔΨm, mitochondrial membrane potential; PINK1, PTEN-induced kinase 1; Parkin, Parkin RBR E3 ubiquitin ligase; LC3, microtubule-associated protein 1A/1B light chain 3; BNIP3, BCL2 interacting protein 3; FUNDC1, FUN14 domain-containing protein 1; NIX, NIP3-like protein X; FIS1, fission protein 1; DRP1, dynamin-related protein 1; MFN2, mitofusin 2; OPA1, optic atrophy protein 1.

After ICH, excessive iron accumulates in mitochondria. Upregulation of SLC25A28 after ICH triggers mitochondrial Fe^2+^ influx and overload [60] (Fig. 3A). Additionally, Fe^2+^ can enter mitochondria through organelle MCSs, which will be described in subsequent sections. In a neonatal rat embryonic matrix hemorrhage model, FTMT is adaptively upregulated to buffer excess iron in mitochondria [61,62]. During ferroptosis, opening of voltage-dependent anion channel 1 (VDAC1) on the outer mitochondrial membrane, is associated with increased mitochondrial membrane potential (ΔΨm), excessive ROS generation, and Ca^2+^ overload, culminating in ATP loss [63,64]. Consistent with this mechanism, VDAC1 expression is increased in neonatal cerebral hemorrhage models [62]. In parallel, excessive Fe^2+^ has been reported to activate the mitochondrial calcium uniporter (MCU), which is also upregulated after ICH, driving substantial Ca^2+^ influx into the mitochondrial matrix [[65], [66], [67]]. MCSs facilitate Ca^2+^ entry too, which will be discussed in ER section. The mitochondrial permeability transition pore (mPTP) is a multiprotein complex situated at the interface between the inner and outer mitochondrial membranes. Elevated Ca^2+^ promotes mPTP opening through binding sites located between the α and β subunits of the F_1_ sector of ATP synthase [68]. Opening mPTP compromises respiratory chain function and increases mitochondrial ROS (mtROS) generation, with complex I being a prominent source [69]. Mitochondrial DNA (mtDNA) lacks histone protection and is particularly vulnerable to ROS, resulting in impaired mitochondrial gene expression, reduced activity of respiratory chain complexes, ultimately diminished efficiency of ATP production [[70], [71], [72]]. In line with it, brain tissue from ICH patients exhibits markedly impaired mitochondrial oxidative metabolism and reduced oxygen consumption in perihematomal regions, indicating profound disruption of energy metabolism [73].

#### 4.1.2 Mitochondria biogenesis

When mitochondrial injury results in inadequate ATP supply, cells may compensate by increasing mitochondrial mass and restoring functional competence, a process referred to as mitochondrial biogenesis [74] (Fig. 3B). Sirtuin 1 (SIRT1), an NAD^+^-dependent protein deacetylase, facilitates mitochondrial biogenesis by deacetylating peroxisome proliferator-activated receptor-γ coactivator 1α (PGC-1α) [75]. Activated PGC-1α induces nuclear respiratory factor 1 (NRF1), which in turn upregulates mitochondrial transcription factor A (TFAM). TFAM translocates to mitochondria, binds and promotes mtDNA transcription and replication, thereby supporting mitochondrial renewal [74,76]. In brain tissue after ICH, total SIRT1 expression increases, whereas nuclear SIRT1 is reduced, a shift that is associated with impaired mitochondrial biogenesis [[75], [76], [77], [78]]. An early compensatory rise in PGC-1α appears to partially restore mitochondrial biogenesis during the acute phase [[75], [76], [77], [78]]. Pharmacological activation of GPR39 enhances mitochondrial biogenesis after ICH via the cAMP response element-binding protein (CREB) and PGC-1α axis [78]. Likewise, HLY78 has been reported to promote mitochondrial biogenesis after ICH through the LRP6, GSK3β, SIRT1, and PGC-1α signaling cascade, thereby attenuating downstream oxidative stress [77]. AMP activated protein kinase (AMPK) further amplifies PGC-1α activity through two complementary routes, including increasing intracellular NAD^+^ levels to augment SIRT1 activity and directly phosphorylating PGC-1α [79,80]. Beyond biogenesis, PGC-1α also enhances antioxidant defenses by inducing NRF2 and peroxisome proliferator-activated receptor-α (PPARα) signaling [81,82]. Moreover, PGC-1α upregulates uncoupling protein 2 (UCP2), which partially uncouples oxidative phosphorylation from ATP synthesis and limits mtROS generation [83]. In ICH, UCP2 activation reduces mitochondrial oxidative stress and suppresses ferroptosis [84].

#### 4.1.3 Mitophagy

Mitophagy is a selective autophagic process that removes dysfunctional mitochondria through the autophagic flux. After ICH, mitochondrial insults including excessive mtROS production, mtDNA damage, and loss of ΔΨm can initiate mitophagy [85] (Fig. 3C). In the ubiquitin-dependent pathway, PTEN-induced kinase 1 (PINK1) accumulates on the outer mitochondrial membrane and recruits parkin RBR E3 ubiquitin protein ligase (Parkin). Ubiquitin adaptors such as optineurin (OPTN) then couple ubiquitinated cargo to the microtubule-associated protein 1A/1B light chain 3 (LC3) machinery, enabling phagophore recruitment, cargo engulfment, and subsequent lysosomal degradation [85]. In receptor mediated mitophagy, outer membrane receptors including BCL2-interacting protein 3 (BNIP3) and NIP3-like protein X (NIX) as well as FUN14 domain-containing 1 (FUNDC1) directly engage LC3, thereby promoting lysosomal clearance of damaged mitochondria [85]. Increased expression of BNIP3, PINK1, Parkin, and FUNDC1 has been reported, typically peaking within 3 days, and mitophagy structures have been observed by TEM after ICH [86,87]. Notably, the literature is not entirely consistent. One study documented a time-dependent reduction in PINK1 protein levels during the first 3 days after ICH [88], whereas clinical observations indicate that PINK1 protein is decreased in ICH patients despite elevated PINK1 mRNA [89]. Functionally, mitophagy is often viewed as a protective response that limits ferroptosis after ICH by clearing damaged iron-containing mitochondria and reducing mtROS production. Nonetheless, excessive mitophagy may also shift iron handling in a manner that ultimately increases iron availability and promotes LPO, thereby facilitating ferroptosis [85].

#### 4.1.4 Mitochondrial dynamics imbalance

After ICH, mitochondrial dynamics shift toward fragmentation, characterized by enhanced fission, impaired fusion, and a concurrent increase in ferroptosis vulnerability (Fig. 3D). Fission protein 1 (FIS1), an outer mitochondrial membrane component, is upregulated after ICH [84,90]. By facilitating recruitment of dynamin-related GTPase protein 1 (DRP1) to mitochondria, FIS1 promotes mitochondrial membrane scission and drives fission [84,90]. Consistently, ICH is associated with increased DRP1 phosphorylation at Ser616 and reduced phosphorylation at Ser637, a modification pattern that favors mitochondrial fragmentation [91,92]. DRP1 knockout induces mitochondrial elongation, attenuates ferroptosis associated loss of ΔΨm, and stabilizes Fe^2+^ transport and storage [93]. A bidirectional relationship between DRP1-dependent fission and ferroptosis has been reported. On the one hand, ferroptosis stimuli promote mitochondrial fragmentation through DRP1 activation [94,95]. On the other hand, DRP1 mediated fission can further facilitate ferroptosis [96]. Interestingly, fragmented mitochondria may also support fatty acid catabolism and are associated with increased GPX4 expression, suggesting a potential compensatory adaptation of ferroptosis [97,98]. Mitofusin (MFN) is a GTPase that mediates outer membrane fusion. MFN2 downregulation in ICH exacerbates ferroptosis. Conversely, its overexpression antagonizes DRP1 driven fission and delays ferroptosis progression [84,93,94]. Optic atrophy protein 1 (OPA1), a key regulator of inner membrane fusion, is also reduced after ICH, which contributes to mitochondrial fragmentation. Restoration of OPA1 expression promotes mitochondrial fusion and improves neurological outcomes after ICH through reestablishing mitochondrial homeostasis and suppressing ferroptosis [99,100]. However, one study proposed that OPA1 GTPase activity promotes ferroptosis independently of mitochondrial fusion, and OPA1 deletion reduced mitochondrial ROS production while inducing ATF4-dependent activation of the system Xc^−^/GSH/GPX4 axis, thereby limiting ferroptosis [101].

Collectively, mitochondria are exposed to diverse forms of injury after ICH, biasing cells toward ferroptosis.

### 4.2 Endoplasmic reticulum

**Fig. 4:**
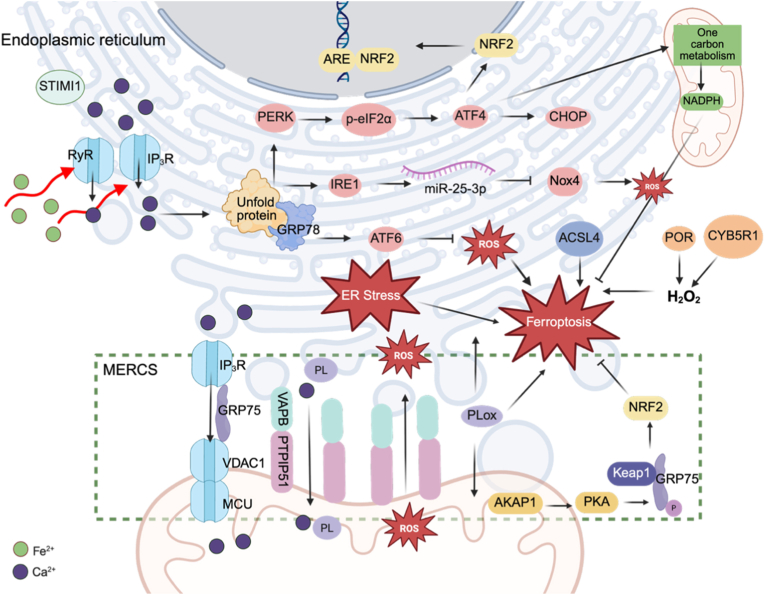
ER stress and mitochondria-ER contacts in ferroptosis after ICH. Fe^2+^ overload activates IP_3_Rs and RyRs, causing ER Ca^2+^ release to the cytosol and mitochondria and triggering ER stress. PERK induces *p*-eIF2α-ATF4 and NRF2, whereas sustained signalling is associated with CHOP. IRE1α induces miR-25-3p to suppress Nox4 translation. ATF6 limits oxidative stress. ER-enriched ACSL4 promotes PUFA-phospholipid formation, and ER oxidoreductases use NADPH to generate ROS that drive LPO. MERCSs coordinate Ca^2+^ and lipid exchange via VAPB-PTPIP51 and the IP_3_R-GRP75-VDAC1 complex, facilitating Ca^2+^ transfer, mtROS and PLox trafficking. MERCS-associated AKAP1-PKA phosphorylates GRP75 to further stabilize NRF2 and restrains ferroptosis. Abbreviations: STIM1, Stromal interaction molecule 1; IP3R, inositol 1,4,5-trisphosphate receptor; GRP78, glucose-regulated protein 78 kDa; PERK, protein kinase R-like ER kinase; ATF6, activating transcription factor 6; IRE1α, inositol-requiring enzyme 1α; eIF2α, eukaryotic initiation factor 2α; *p*-eIF2α, phosphorylated eIF2α; miR-25-3p, microRNA-25-3p; NOX4, NADPH oxidase 4; CHOP, CCAAT/enhancer-binding protein homologous protein; ATF4, activating transcription factor 4; POR, cytochrome P450 reductase; CYB5R1, cytochrome *b*5 reductase 1; MERCS, mitochondria-ER contact sites; GRP75, glucose-regulated protein 75; PTPIP51, protein tyrosine phosphatase interacting protein 51; VAPB, VAMP-associated protein B; PLox, oxidized phospholipids; PKA, protein kinase A; RyR, ryanodine receptor; AKAP1, A-kinase anchoring protein 1.

ER is a multifunctional organelle that supports protein folding, trafficking, and post translational modification, and serves as a major site for Ca^2+^ storage, lipid biosynthesis, and carbohydrate metabolism. Stromal interaction molecule 1 (STIM1) is a calcium sensor primarily localized to the endoplasmic reticulum membrane. After ICH, STIM1 expression is upregulated and promotes iron influx by interacting with TFR1 at the plasma membrane, thereby enhancing TFR1 activity [102]. Fe^2+^ overload perturbs ER Ca^2+^ homeostasis and induces ER stress (Fig. 4). Mechanistically, excess Fe^2+^ activates ER Ca^2+^ release channels, including inositol 1,4,5- trisphosphate receptors (IP_3_Rs) and ryanodine receptors (RyRs), resulting in substantial Ca^2+^ leakage from ER into the cytosol and mitochondria [[103], [104], [105]]. Disrupted Ca^2+^ handling, together with oxidative stress, promotes the accumulation of unfolded or misfolded proteins within the ER lumen, a condition collectively referred to as ER stress [106,107]. To restore proteostasis, cells engage the unfolded protein response (UPR), which is initiated by three principal ER transmembrane sensors: inositol-requiring enzyme 1 alpha (IRE1α), protein kinase R-like ER kinase (PERK), and activating transcription factor 6 (ATF6) [108]. Glucose-regulated protein 78 kDa (GRP78) is a key ER chaperone that maintains these sensors in an inactive state under basal conditions. Upon ER stress, GRP78 dissociates from PERK, IRE1α, and ATF6, enabling their activation [108]. The PERK branch is rapidly engaged in the early phase, approximately 12 h after ICH, with marked increases in phosphorylated eukaryotic initiation factor 2α (*p*-eIF2α) and activating transcription factor 4 (ATF4) [109,110]. ATF4 enhances antioxidant defenses by inducing NRF2, thereby suppressing ferroptosis [[111], [112], [113], [114]]. When stress exceeds adaptive capacity, persistent ER stress can instead exacerbate ferroptosis and may trigger CCAAT/enhancer-binding protein homologous protein (CHOP) mediated apoptosis [110,[115], [116], [117], [118], [119]]. GRP78 and CHOP protein levels rise markedly after ICH and peak around 7 days, coinciding with enhanced ferroptosis activity [120,121]. Beyond canonical UPR outputs, the PERK and ATF4 axis has also been reported to support mitochondrial function by transcriptionally activating genes involved in mitochondrial one-carbon metabolism, thereby increasing NADPH generation, while restraining excessive GRP78, PERK, and ATF4 signaling [110]. In parallel, phosphorylation of IRE1α (p-IRE1α) is upregulated after ICH and suppresses Nox4 translation through induction of miR-25-3p, which lowers ROS production [122]. Upregulation of ATF6 after ICH likewise alleviates oxidative stress and promotes M2 polarization of microglia [123]. Accumulating evidence suggests that, during ferroptosis, membrane LPO is initiated and preferentially accumulates within the ER before spreading to mitochondria and, ultimately, the plasma membrane, a process that may involve ER resident proteases [[124], [125], [126]]. Supporting this view, ACSL4 is predominantly enriched in the ER [127]. Moreover, ER localized oxidoreductases, including cytochrome P450 reductase (POR) and cytochrome *b*5 reductase 1 (CYB5R1), have been shown to use NADPH to generate ROS, thereby promoting membrane LPO [[128], [129], [130]].

Mitochondria-ER contact sites (MERCSs), also referred to as ER-mitochondria contact sites (EMCSs) or mitochondria-associated ER membranes (MAMs), are specialized microdomains that coordinate Ca^2+^ signaling and lipid exchange [131], although their net impact on ferroptosis remains debated (Fig. 4). Protein tyrosine phosphatase-interacting protein 51 (PTPIP51), also known as regulator of microtubule dynamics protein 3 (RMDN3), is an outer mitochondrial membrane protein that interacts with VAMP-associated protein B (VAPB), an ER membrane protein, to form a tethering complex that supports ER-to-mitochondria transfer of Ca^2+^ and phospholipids [132,133]. In addition, an IP_3_R, glucose-regulated protein 75 (GRP75), and VDAC1 complex at MERCSs provides another route for regulating Ca^2+^ flux [134]. Under ferroptosis stimuli, Ca^2+^ enter mitochondria through MERCSs, promoting mtROS generation and subsequent LPO [135]. Recent work further proposes that MERCSs may represent an early platform for LPO. Within minutes after ferroptosis induction, oxidized phospholipids (PLox) become detectable at MERCSs, followed by rapid expansion of MERCSs and transfer of PLox to mitochondria. Disrupting MERCSs limits mitochondrial exposure to lipid derived ROS and confers protection [135,136]. One mechanistic explanation is that impaired MERCSs-dependent phospholipid transport reduces ER mitochondria lipid coupling, favoring accumulation of PUFA-containing triacylglycerols (TAGs). They can be sequestered into LDs, decreasing their availability for ferroptosis PLox [135,137]. Interestingly, other observations support a protective role for MERCSs. mtROS has been reported to increase formation of the VAPB-PTPIP51 tethering complex, facilitating transfer of ROS, L•, and LPO from mitochondria toward the ER, thereby relieving mitochondrial oxidative stress and supporting cell survival [138]. Moreover, MERCSs can locally scaffold A-kinase anchoring protein 1 (AKAP1) and protein kinase A (PKA), enabling phosphorylation of GRP75 at Ser148, which promotes GRP75 relocalization and interaction with Keap1, stabilizing NRF2 and suppressing ferroptosis [126]. GRP75 expression is decreased in ICH, whereas GRP75 overexpression reduces inflammation and may also attenuate neuronal apoptosis [139].

### 4.3 Lysosomes

**Fig. 5:**
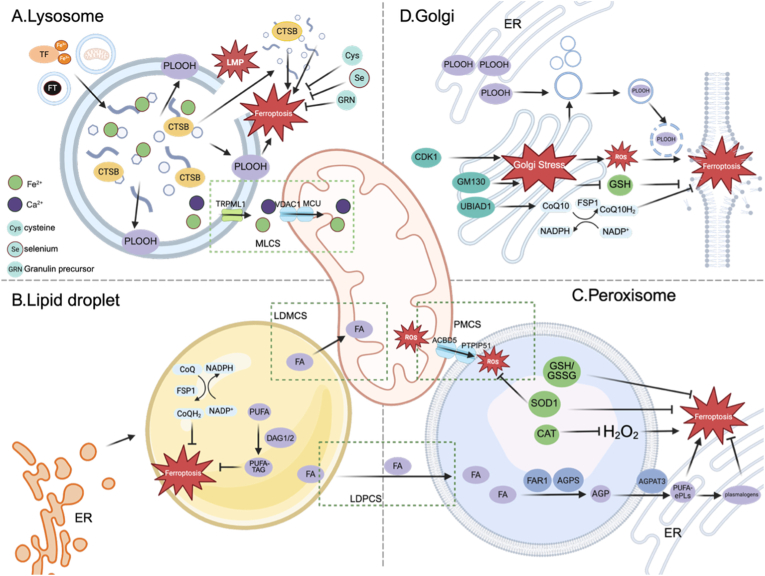
Lysosome, lipid droplet, peroxisome and the Golgi in ferroptosis after ICH. (A) Lysosome. Lysosomes concentrate Fe^2+^ and lipid peroxides. Increased lysosomal membrane permeability releases Fe^2+^ and CTSB to amplify oxidative damage and ferroptosis. Lysosomal TRPML1 at LDMCS transfers Ca^2+^ and Fe^2+^ to mitochondrial. (B) Lipid droplet. DGAT1/2-driven PUFA sequestration into TAG reduces oxidizable membrane substrates, whereas LD-localized FSP1-CoQ10 buffers neutral-lipid peroxidation. (C) Peroxisome. Ether-lipid synthesis (FAR1-AGPS-AGPAT3) supplies PUFA-ePLs, which feed into ferroptosis sensitivity. Peroxisomes generate H_2_O_2_ but counterbalance it with CAT and SOD1. PMCS enable ROS transfer. (D) Golgi. Golgi stress is associated with ROS accumulation. LPO species traffic from the ER to Golgi vesicles and propagate oxidative injury. UBIAD1 supports CoQ10 generation that cooperates with FSP1 to restrain lipid peroxidation. Abbreviations: CTSB, cathepsin B; LMP, lysosomal membrane permeability; TRPML1, transient receptor potential mucolipin 1; GRN, granulin precursor; DGAT1/2, diacylglycerol acyltransferase 1/2; TAG, triacylglycerol; SOD1, superoxide dismutase 1; FA, Fatty acid; FAR1, fatty acyl-CoA reductase 1; AGPS, alkyldihydroxyacetonephosphate synthase; AGPAT3, 1-acylglycerol-3-phosphate O-acyltransferase 3; PUFA-ePLs, PUFA-containing ether phospholipids; ACBD5, acyl-CoA-binding domain-containing 5; CDK1, cyclin-dependent kinase 1; GM130, Golgi matrix protein 130; UBIAD1, UbiA prenyltransferase domain-containing protein 1; LDMCS, LD-mitochondrial contact site; LDPCS, LD-peroxisome contact site; PMCS, peroxisome-mitochondrial contact site.

Lysosomes serve as a hub for post-phagocytic hemoglobin processing and integrate iron inputs from transferrin endocytosis, ferritinophagy, mitophagy and intracellular iron stores [[140], [141], [142]] (Fig. 5A). YTHDC2 expression is markedly reduced after ICH, which is accompanied by increased NCOA4 expression, enhanced ferritinophagy, and a consequent facilitation of ferroptosis [143]. Several studies further propose that early ferroptosis events preferentially arise within lysosomes [144,145]. For example, the GPX4 inhibitor RSL3 initiates membrane LPO in lysosomes, and LPO remains largely confined to lysosomes during the first hour [[144], [145], [146]]. As lysosomal membrane permeability (LMP) increases, Fe^2+^ and lysosomal proteases leak into the cytosol, providing a route for amplification and dissemination of LPO signaling to the ER and other organelles [[144], [145], [146]]. Among lysosomal proteases, cathepsin B (CTSB) has been implicated in ferroptosis through histone H3 cleavage or induction of DNA damage [147,148]. After ICH, increased m6A modification of CTSB transcripts enhances CTSB translation, resulting in CTSB upregulation and promoting microglial ferroptosis [149]. Although lysosomes are commonly viewed as pro-ferroptosis organelles, some lysosome-derived metabolites and transport processes can also restrain ferroptosis. Lysosome-dependent availability of cysteine and selenium has been linked to ferroptosis resistance in certain contexts [[150], [151], [152]]. In addition, granulin precursor (GRN) derived from microglial lysosomes suppresses ferroptosis associated neuronal injury by inducing NRF2 expression [150,153].

Mitochondria-lysosome contact sites (MLCSs) regulate mitochondrial Ca^2+^ handling through the lysosomal Ca^2+^ release channel transient receptor potential mucolipin 1 (TRPML1). TRPML1-dependent Ca^2+^ efflux from lysosomes can be coupled to mitochondrial Ca^2+^ entry, a process coordinated by MLCSs together with VDAC1 and MCU [154]. Beyond Ca^2+^ signaling, TRPML1 has also been implicated in Fe^2+^ transfer from lysosomes to mitochondria, and pharmacological or genetic inhibition of TRPML1 alleviates mitochondrial Fe^2+^overload [155]. In line with it, TRPML1 protein levels are elevated in brain tissue after ICH, whereas TRPML1 downregulation mitigates severe brain injury [156].

### 4.4 Lipid droplets

LDs contain a neutral lipid core, mainly TAGs and sterol esters, surrounded by a phospholipid monolayer. As dynamic organelles, LDs buffer cellular lipid availability by storing and mobilizing lipids in response to metabolic demand [157,158]. In ferroptosis, PUFAs are intrinsically more susceptible to peroxidation than MUFAs, shifting membrane composition toward a higher MUFA-to-PUFA ratio can lower ferroptosis vulnerability [157]. Esterifying PUFAs into TAGs via diacylglycerol acyltransferase 1/2 (DGAT1/2) and sequestering these neutral lipids within LDs can further limit LPO [159] (Fig. 5B). Antioxidants in LDs may also contribute to ferroptosis control. The LD-localized FSP1/CoQ10 system suppresses peroxidation of neutral lipid, providing a plausible explanation for the anti-ferroptosis effect of LDs sequestration [160]. Conversely, in the absence of FSP1, PUFA-enriched LDs permit triglyceride peroxidation and promote ferroptosis [160]. However, abnormal accumulation of LDs in perihematomal tissue has been linked to heightened neuroinflammation and worsened neurological dysfunction [161,162], suggesting that LD antioxidant capacity may become dysregulated after ICH, although the underlying mechanisms remain to be defined.

LD-mitochondrial and LD-peroxisome contact sites (LDMCSs and LDPCSs) constitute a key structural basis for directional intracellular fatty acid trafficking and metabolic coupling. Under nutrient deprivation or starvation stress, these contacts are reinforced through multiple mechanisms, promoting fatty acid transfer from LDs to mitochondria and peroxisomes [[163], [164], [165], [166]]. Consistent with this spatial coupling, during PUFA- and Fe^2+^-driven ferroptosis, PUFA-loaded LDs positioned closer to mitochondria or peroxisomes undergo ferroptosis earlier [167]. Nonetheless, how these MCSs shape ferroptosis specifically in ICH remains unknown.

### 4.5 Peroxisomes

Peroxisomes are ubiquitous single-membrane organelles and have recently emerged as newly identified participants in ferroptosis (Fig. 5C). However, how peroxisomes contribute to ferroptosis after ICH remains unknown. Peroxisomes are enriched in oxidases that generate H_2_O_2_. To limit oxidative injury, mammalian peroxisomes harbor an array of antioxidant enzymes, including catalase (CAT), superoxide dismutase 1 (SOD1), and others [168]. Notably, GSH and oxidized glutathione (GSSG) can freely diffuse across the mammalian peroxisomal membrane. Even under CAT deficiency, when H_2_O_2_ level rises in peroxisomes, GSH remains capable of protecting proteins against oxidative modification [168]. Moreover, peroxisomes are the initiation of ether phospholipid (e-PL) biosynthesis [169]. Within peroxisomes, fatty acyl-CoA reductase 1 (FAR1), glyceronephosphate O-acyltransferase (GNPAT), and alkylglycerone phosphate synthase (AGPS) cooperatively generate the ether-lipid precursor 1-*O*-alkyl-glycerol-3-phosphate (AGP), which is then transported to the ER and, with the involvement of 1-acylglycerol-3-phosphate O-acyltransferase 3 (AGPAT3), incorporates PUFAs to form PUFA-ePLs [170]. These PUFA-ePLs may be converted into antioxidant plasmalogens, but they can also act as substrates for lipid peroxidation [170,171]. Accordingly, disrupting peroxisome biogenesis or e-PL synthesis reduces ferroptosis sensitivity in some contexts [170,172,173].

ROS exchange at peroxisome-mitochondrial contact sites (PMCSs) exerts a direct influence on mitochondrial redox homeostasis. Recent work indicates that mitochondrial oxidative stress promotes the formation of PMCSs between the peroxisomal membrane protein acyl-CoA-binding domain-containing 5 (ACBD5) and PTPIP51, facilitating ROS transfer from mitochondria into peroxisomes [174].

### 4.6 Golgi apparatus

The Golgi apparatus is central to protein processing and vesicular trafficking and contributes to lipid metabolism. Disruption of Golgi architecture causes Golgi stress, which is accompanied by increased intracellular ROS, depletion of GSH, and ultimately ferroptosis [175]. In ICH models, upregulation of cyclin-dependent kinase 1 (CDK1) and the Golgin subfamily A member 2 (GM130) has been linked to loss of Golgi integrity and induction of Golgi stress responses [[176], [177], [178]] (Fig. 5D). Mechanistically, the earliest LPO signals during ferroptosis are reported to arise at ER, after which PLOOH accumulate within Golgi-associated vesicular structures [179]. Vesicle rupture then functions as a trigger that disseminates LPO chain reactions across other membrane systems, with oxidative damage ultimately converging at the plasma membrane [179].Within the Golgi, UBIAD1 mediates CoQ10 biosynthesis and cooperates with FSP1 to restrain LPO [50]. After ICH, UBIAD1 expression declines, whereas L-3-*n*-butylphthalide treatment restores UBIAD1 levels and improves neurological outcomes [180].

## 5 Potential targets of ferroptosis after ICH

Although organelle-specific changes during ferroptosis have been characterized in depth, substantial knowledge gaps persist in ICH. We summarized candidate therapeutic strategies across organelles described in the preceding sections (Table 1). Nevertheless, key mechanistic uncertainties and translational barriers remain. We therefore collate recent findings to delineate potential anti-ferroptosis targets of ferroptosis after ICH.

**Table 1:** Anti-ferroptosis targets across organelles.

Organelle	Intervention	Target	Mechanism	Effect	Stage
Mitochondria	SRT1720 [75]	SIRT1	Nuclear SIRT1 ↑, deacetylation of PGC-1α↑, **mitochondria biogenesis** ↑, mitochondrial electron transport chain proteins ↑, apoptotic proteins ↓.	Mortality ↓, neurobehavioral deficits ↓, brain water content ↓.	Preclinical
	GPR39 [78]	PGC-1α	Activate CREB/PGC-1α pathway, **mitochondria biogenesis** ↑.	Brain edema ↓, hematoma size ↓, neuronal degeneration ↓, neuronal death ↓, neurobehavioral deficits ↓.	Preclinical
	HLY78 [77]	PGC-1 α	*p*-LRP6 ↑,p-GSK3β ↑, Sirt1 ↑, PGC-1α ↑, **mitochondria biogenesis ↑**,Romo-1 ↓, C-Caspase-3 ↓.	Neurobehavioral deficits ↓, oxidative stress ↓, neuronal apoptosis ↓.	Preclinical
	B355252 [84]	UCP2	Activate UCP2, MFN2 ↑, FIS1 ↓, **mitochondria fusion ↑**.	hematoma ↓, ROS ↓, ferroptosis ↓, neurobehavioral deficits ↓.	Preclinical
	PINK1 overexpression [89]	PINK1	**Mitophagy ↑**	Neurobehavioral deficits ↓.	Preclinical
	Hydralazine [91]	Acrolein	Acrolein ↓, DRP1 translocation to mitochondrial membrane ↓, **mitochondria fission ↓**.	Neural apoptosis ↓, brain edema ↓, neurobehavioral deficits ↓.	Preclinical
	Recombinant APN peptide (APNp) [92]	DRP1	DRP1 serine 637 (S637) phosphorylation ↑, translocation of DRP1 to the mitochondrial membrane ↓, **mitochondria fission ↓**.	Inflammatory brain injury ↓.	Preclinical
	ATP synthase coupling factor 6 (ATP5J) knock down [90]	DRP1, FIS1	DRP1 ↓, FIS1 ↓, **mitochondria fission ↓**, mPTP opening ↓ , mitochondrial respiratory electron transport chain activity ↑.	BBB disruption ↓, brain water content ↓, ROS↓, neurobehavioral deficits ↓.	Preclinical
	Celastrol [99]	OPA1	OPA1 ↑, **mitochondria fusion ↑**.	Oxidative stress ↓, neurobehavioral deficits ↓, neuronal apoptosis ↓.	Preclinical
	Pterostilbene [100]	OPA1	OPA1 ↑, **mitochondria fusion ↑**.	Microglial inflammation ↓, BBB damage ↓, brain edema ↓, neurobehavioral deficits ↓.	Preclinical
ER	S–IN–1 [103]	STIM1-specific inhibitor targeting the Lys385 site	The affinity and interaction between STIM1 and TFR1 ↓, neuronal iron accumulation ↓, ferroptosis ↓.	Neuronal viability **↑**, brain tissue damage ↓,neurobehavioral deficits ↓.	Preclinical
	Tauroursodeoxycholic acid (TUDCA) [120]	GRP78	GRP78 ↓, **ERS ↓**.	Neuronal degradation ↓, neurobehavioral deficits ↓.	Preclinical
	Didang Tang [121]	PERK	Inhibition of PERK, GRP78 ↓, eIF2α↓, ATF4↓, CHOP↓, GPX4 ↑, SLC7A11 ↑, **ERS ↓**.	Hematoma ↓, iron deposition ↓, MDA ↓, ferroptosis ↓, neurobehavioral deficits ↓.	Preclinical
	Salubrinal [109]	eIF2α	*p*-eIF2α ↑, ATF4 ↑, **ERS ↑**(early stage of ICH).	Neuronal survival ↑, apoptosis↓.	Preclinical
Lysosome	YTHDC2 overexpression [143]	NCOA4	m6A modification of NCOA4 mRNA, **ferritinophagy ↓**, FTH ↑, FTL ↑, Fe^2+^ ↓, GSH ↑.	Brain water content ↓, neuronal death ↓, MDA ↓, ferroptosis ↓, neurobehavioral deficits ↓.	Preclinical
	CA-074, Ctsb knock down [149]	Cathepsin B	Cathepsin B ↓, ACSL4 ↓, GPX4 ↑.	LPO ↓, ROS ↓, MDA ↓, Ferroptosis ↓.	Preclinical
	TRPML1 knock down [156]	TRPML1	TRPML1 ↓	Inflammatory cytokines ↓, neuronal cell death and degeneration ↓, learning and memory ↑.	Preclinical
Golgi	L-3-*n*-butylphthalide (NBP) [180]	UBIAD1	UBIAD1 ↑, apoptotic cells ↓, neurological deficit ↓.	Cell apoptosis ↓, neurobehavioral deficits ↓.	Preclinical

Initially characterized as a plasma membrane associated anti-ferroptosis factor, FSP1 was subsequently detected within a perinuclear membrane compartment, partially colocalizing with ER and Golgi [57]. More recently, FSP1 has also been shown to localize to LDs, where it preserves neutral lipid integrity and limits the accumulation of oxidized lipids [160]. These observations collectively suggest that FSP1 operates as a multi-organelle antioxidant system. In addition to forming an antioxidant system with CoQ10, vitamin K (VK) represents another key redox partner. FSP1 reduces vitamin K (VK) to vitamin K hydroquinone (VKH_2_) in an NAD(P)H-dependent manner, thereby limiting LPO and suppressing ferroptosis [181]. Given the pivotal role of FSP1-mediated defense and the relatively limited assessment of its therapeutic relevance in ICH, this pathway merits renewed investigation. Omarigliptin (MK3102) has been reported to restore FSP1 expression after ICH [59]. Idebenone, a CoQ10 analog, enhances mitochondrial electron transport and has also been shown to stabilize FSP1 to inhibit ferroptosis [182,183]. In ICH, idebenone attenuates brain injury and inflammation, although whether these effects are directly mediated through FSP1 was not examined [184]. Additional antioxidant programs can also suppress ferroptosis, although none have yet been reported in ICH. Dihydroorotate dehydrogenase (DHODH), situated on the mitochondrial inner membrane, curtails LPO chain propagation by channeling electrons to CoQ10 and thereby producing reduced CoQ10H_2_ [185]. Meanwhile, GTP cyclohydrolase 1 (GCH1) acts as a rate-limiting enzyme that catalyzes the synthesis of tetrahydrobiopterin (BH4) [186]. BH_4_ can directly quench PLOO• within membranes, interrupting the ferroptosis LPO cascade, and this axis functions independently of GPX4 activity [186,187].

MCSs are increasingly implicated in ferroptosis because of privileged microdomains for inter-organelle signaling and metabolite-exchange. However, how MCSs contribute to ferroptosis in ICH remains largely undiscovered. Several reports nonetheless suggest that ferroptosis is accompanied by enhanced MAMs, and that physical isolation or disruption of them can dampen ferroptosis. Mechanistically, MCSs facilitate Ca^2+^ transfer to mitochondria. For example, Glycogen synthase kinase 3 beta (GSK3β) phosphorylates IP_3_Rs, thereby enhancing Ca^2+^ flux from ER into mitochondria. Inhibition of GSK3β with SB216763 diminishes IP_3_R phosphorylation, restrains mitochondrial Ca^2+^ overload, and limits mPTP opening [188,189]. Likewise, VBIT-4 and VBIT-12 blunt ER-to-mitochondria Ca^2+^ transfer through inhibition of VDAC1 [135]. The GRP75 inhibitor MKT-077 reduces MAM abundance and disrupts IP_3_R-VDAC1 coupling, which preserves mitochondrial function and Ca^2+^ homeostasis [190]. Sigma-1 receptor (σ1R), a chaperone enriched at MAMs, has been implicated as a key modulator of ferroptosis execution [135]. CGI1746, identified from a kinase-inhibitor screen, targets σ1R and suppresses ferroptosis by disrupting MAMs [135]. Conversely, several studies argue that reinforcing MCSs can redistribute oxidative stress and LPO. The REEP5-MFN1/2 complex has been identified as an ER-mitochondria tether, and REEP5 deficiency tends to increase mtROS, whereas enforced dimerization strengthens contacts and reduces mtROS [191]. mtROS can promote increased MERCSs or PMCSs, enabling mtROS transfer to the ER or peroxisomes [138,174]. In this framework, enhancing specific tether linkages, such as PTPIP51 with ACBD5 or VAPB, emerges as a promising intervention.

Finally, intracellular propagation of LPO is not necessarily limited to MCSs routes. The Golgi has historically been underemphasized in ferroptosis research. However, Xu et al. reported that Golgi-associated vesicles can disseminate LPO to other organelles and plasma membrane, indicating a vesicle-based, contact-independent mode of LPO transmission [179]. Therefore, it is of great significance to explore how lipid-binding proteins shape Golgi-associated vesicles and regulate intracellular lipid transport.

## 6 Concluding remarks and future perspectives

ICH remains a major cause of death among cerebrovascular disorders. Clarifying its pathophysiology is essential for designing effective protective strategies. Hematoma resolution comes at the cost of iron release. Ferroptosis has emerged as a pivotal contributor to SBI after ICH, which engages multiple organelles. The ER, lysosomes, and MERCSs have each been proposed as initial sites of LPO [124,125,136,145]. MCSs are major conduits for intracellular trafficking of LPO, Fe^2+^, and ROS, while also help oxidative stress redistribute among organelles. Mechanistic exploration on ferroptosis specifically after ICH remains limited. Current anti-ferroptosis strategies after ICH have largely centered on iron chelation and activation of the NRF2 axis, whereas the translational value of alternative pathways has received comparatively little attention. Future research should aim to elucidate the molecular mechanisms of ferroptosis and how organelles engaged in, explore more refined therapeutic strategies, and integrate the advantages of multiple therapies to develop a comprehensive treatment strategy for ICH.

## Declaration of generative AI use

The authors used ChatGPT (OpenAI) to improve language and grammar refinement of the manuscript. No AI tools were used for writing, interpretation, or figure generation. The authors are fully responsible for the content.

## Funding sources

This work was supported by grants from 10.13039/501100001809National Natural Science Foundation of China (82371302), Beijing Scholar (097), Noncommunicable Chronic Diseases-10.13039/501100018537National Science and Technology Major Project (2025ZD0552601).

## CRediT authorship contribution statement

**Na Zhou:** Software, Writing – original draft, Writing – review & editing. **Yang Du:** Methodology, Supervision, Writing – review & editing. **Yinuo Wang:** Investigation, Methodology, Writing – review & editing. **Xingquan Zhao:** Conceptualization, Funding acquisition. **Yi Ju:** Conceptualization, Funding acquisition, Methodology, Supervision.

## Declaration of competing interest

The authors have declared that no competing interest exists.

## Data Availability

No data was used for the research described in the article.
